# Evaluation of Long-pulsed 1064 nm Nd:YAG Laser-assisted Hair Removal vs Multiple Treatment Sessions and Different Hair Types in Indian Patients

**DOI:** 10.4103/0974-2077.44163

**Published:** 2008

**Authors:** Rachna Mittal, Snehal Sriram, Kamaldeep Sandhu

**Affiliations:** *Kaya Skin Clinic, Chandigarh, India*

**Keywords:** Nd:YAG laser, laser assisted hair removal, Indian skin phototype

## Abstract

**Background:**

Longer wavelength lasers such as Nd: YAG are considered to be the best for darker skin phototypes.

**Objectives:**

The aim of this study was to investigate the safety and efficacy of long-pulsed, 1064 nm Nd:YAG laser-assisted hair removal in relation to multiple treatment sessions and different hair types in Indian patients.

**Method:**

Fifty-nine adult women with skin phototypes IV and V were treated with a long-pulsed Nd: YAG laser (1064 nm, 10 mm spot size, fluence of 30–50 J/cm^2^, pulse duration 15–30 ms). Six consecutive treatment sessions were given at 4–6 week intervals. The modified Ferriman Gallway system of scoring was used to grade the hairs before each treatment session and six weeks after the last therapy. Based on this grading, three main hair types were recognised: thin vellus (Grade 1), intermediate (Grade 2), and terminal hair (Grades 3 and 4). Patients were divided into three groups: achievers who converted to thin vellus hair (Grade I), responders who shifted to a lower grade but were short of reaching grade 1, and failures who did not show any change throughout the six laser sessions.

**Results:**

Six weeks after six laser treatment sessions, the achievers totaled 56%, responders 23%, and failures 20% of the patient population. At the end of the 3^rd^, 4^th^, 5^th^, and 6^th^ sessions, achievers were 5, 15, 25, and 56% respectively of all the patients (*P* < 0.001, which was statistically significant). Achievers for terminal hair were 57.5% while it was 53.8% for intermediate hair (*P* = 0.9, nonsignificant). There were no permanent side effects. There were no failures in the terminal group while nearly 50% of the patients were failures in the intermediate group.

**Conclusion:**

Six multiple laser treatment sessions with a long-pulsed, 1064 nm Nd: YAG laser with contact cooling were found to be safe and effective for hair reduction in Indian patients with both terminal and intermediate hair. The success rate was found to improve with successive sessions. However, terminal hairs responded better than intermediate hairs.

## INTRODUCTION

The field of laser technology has seen major advancements in the last decade, particularly in the area of laser-assisted hair removal. With the advent of longer wavelength, longer pulse durations and efficient cooling devices, the currently available laser machines can now be used safely for all skin phototypes. The two wavelengths which can safely be used for laser hair reduction on darker skin types are the diode (810 nm) and Nd: YAG (1064 nm).[1] In terms of efficacy, the shorter wavelength (diode) laser is generally regarded as more effective because of ‘melanin’s higher absorption value which decreases with increasing wavelength. However, the longer wavelength Nd: YAG laser is considered ideal for treating patients with darker skin, due to reduced scatter and deeper penetration of the laser light.[1,2] Also, shorter pulse durations can be more safely used with the Nd: YAG laser than with the diode. This is an additional advantage when dealing with finer hair with shorter thermal relaxation time (TRT).[3]

The FDA (Food and Drug Administration) has approved both the long pulsed diode and Nd: YAG laser systems for laser-assisted hair removal in darker skin phototypes. However, irrespective of the type of laser used, multiple treatment sessions are required in order to effectively eliminate the hair. It has been well demonstrated in the past that multiple treatment sessions with the Nd: YAG laser are more effective than a single session.[1–9] However, there is limited data regarding its efficacy in multiple sessions, especially more than five, in darker skin types.[2]

Unwanted body hair can represent a severe cosmetic disturbance. In recent years, not only people with hirsuitism and hypertrichosis, but even those with normal distribution of unwanted hair seek long-term laser hair reduction.

There are three basic types of hair on the human body which differ in colour, texture, and diameter. Vellus hair is the very fine, short, nonpigmented hair with a small cross-sectional area. Terminal hair is the coarse, long, pigmented hair with a large cross-sectional area whereas intermediate hair is the hair that is intermediate in length and shaft size.[10]

Taking this into consideration, an attempt was made to assess the efficacy of laser hair reduction in the different types of hair mentioned above as only one such similar study was found in literature after an extensive Medline search. Therefore, the purpose of this study was two-fold: 1) to evaluate the safety and efficacy of a high-energy, long-pulsed, 1064 nm Nd: YAG laser for hair removal in six multiple consecutive sessions and; 2) its role in relation to terminal and intermediate hair in darker skin.

## MATERIALS AND METHODS

A scoring system was devised for the purpose of standardizing the selection of pulse duration and fluence and for assessing the efficacy of laser hair reduction.

So far, the only standardised and widely used system for scoring of hair has been the Ferriman Gallway System of scoring.[9] It is used for the assessment of hirsuitism and considers only thick terminal hair. We made certain modifications to it and the following grades of hair were recognised based upon three factors, including density, thickness, and colour of hair.

Grade 1 →Fine vellus hairGrade 2 →Light brown, fine, low-density hair}intermediate hairGrade 3 →Dark brown, less coarse, low-density}terminal hairGrade 4 →Dark coarse, high-density hair

While tabulating the results, Grade 2 was referred to as intermediate hair, Grades 3 and 4 were considered together as terminal hair.[8]

A total of 59 patients, 18–48 years old, of a mean age of 28 ± 11 years, with facial hair, and who met the inclusion criteria, were treated with 1064 nm, long-pulsed Nd: YAG laser (Coolglide CV, Cutera, Brisbane, CA). The inclusion criteria were grade 2, 3, or 4 type of hair with skin types IV or V. Six consecutive treatment sessions were delivered at 4–6 week intervals. Exclusion criteria included Grade 1 hair, any previous laser treatment to the study area, any gross hormonal dysfunction, waxing, depilation, electrolysis or bleach use within six weeks of entry into the study, a history of keloid scarring, pregnancy, or immunosuppression.

Treatment sites included were chin (21), upper lip (17), sidelocks (11), cheeks (8), and lower lip (2). The most common site was the chin followed by the upper lip and others.

Immediately before each treatment session, hairs were shaved close to the skin using a safety razor. Topical anaesthetic cream (Prilox, Neon Laboratories Ltd., Mumbai, India) was applied 20–30 min before the procedure whenever required. An integrated contact chill tip was used to protect the epidermis during laser irradiation. Adjacent, overlapping 10 mm spots were placed over the treatment areas. Depending on the type of hair grade, *i.e*., 2, 3, and 4 received a fluence and pulse width of 40–45 J/15–20 ms, 35–40 J/20–25 ms, and 30–35 J/25–30 ms respectively [Table 1]. Fluence was increased by 10% at each treatment visit if no side effects were noted from the previous session.

**Table 1:** Characteristics of hair and the laser specification adopted

Hair grade	No. of patients	Pulsed duration (Range)	Mean pulse (ms)	Fluence (Range J/cm^2^)	Mean fluence (J/cm^2^)
2	26	15–20	15	40–45	43
3	20	20–25	20	35–40	40
4	30	25–30	26	30–35	33

Photographs were obtained of all treatment sites before each treatment session and after the sixth laser treatment in the majority of cases. Follow-up was performed before each treatment session and six weeks after the last therapy during which the hair was assessed by the dermatologist. Patients showing any reduction in their hair density, texture, or colour were shifted to the next lower grade.

At the end of the study, patients were divided into three groups:

- Achievers: patients who converted to Grade I signifying successful treatment
- Responders: patients showing conversion to a lower grade but still short of reading grade 1
- Failures: Patients who did not show any change throughout the six laser sessions. Adverse effects were also recorded at each visit.

## RESULTS

**Figure 1:**
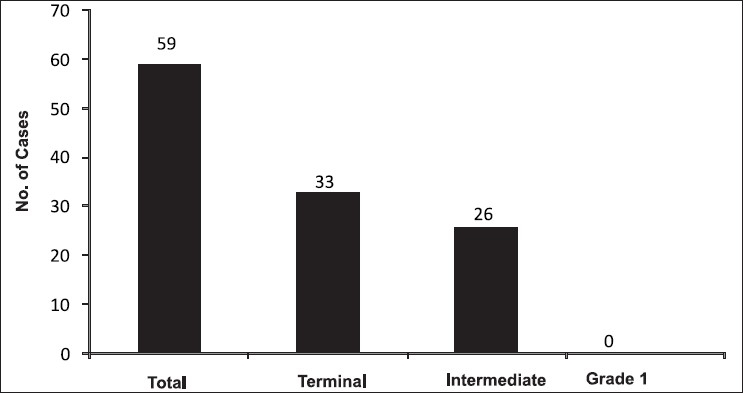
Division into groups according to hair type before laser sessions

At the beginning of the study, the total number of clients was 59 out of which 33 belonged to the terminal group and 26 to the intermediate group, while the number of patients with grade 1 hair was zero as this was the exclusion criterion for the study [Figure 1].

**Figure 2:**
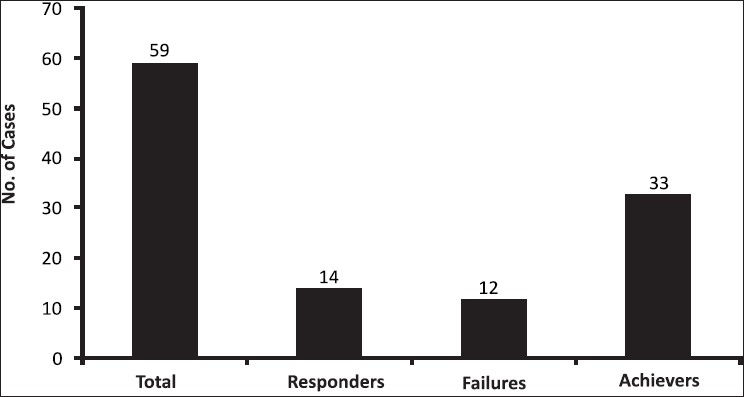
Division into groups based on treatment results after laser sessions

At the end of the study (six weeks following the sixth laser session), the number of achievers signifying successful treatment were 33/59 (56%) [Figure 2].

**Figure 3:**
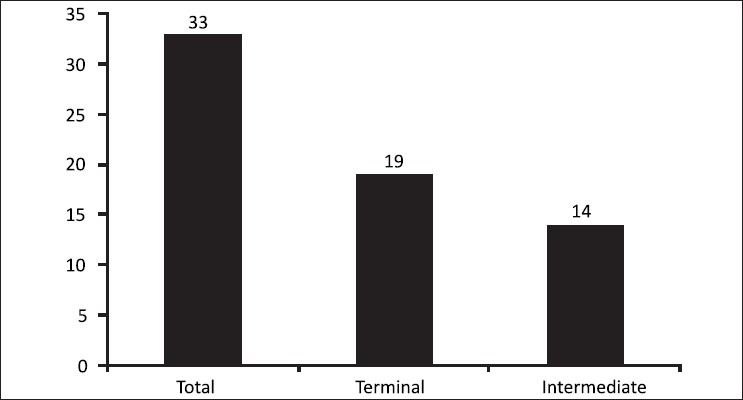
Number of achievers for terminal and intermediate group

Further analysis showed that there were 19/33 (57.5%) achievers for the terminal group while there were 14/33 achievers (53.8%) for the intermediate group. The terminal group had a greater number of achievers although the difference was not statistically significant (*P* = 0.9) [Figure 3].

**Figure 4:**
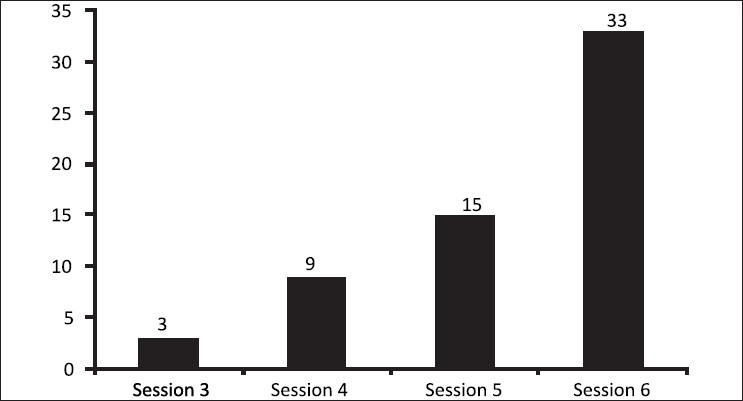
Numbers of achievers with increasing number of laser sessions

The number of achievers tabulated after each individual laser session varied significantly from the 3^rd^ session onwards. The achievers accounted for 5, 15, 25, and 56% for the 3^rd^, 4^th^, 5^th^, and 6^th^ sessions respectively. This dependency between the achievers and the number of treatment sessions was highly significant statistically (*P*< 0.0001) [Figure 4].

There were 14/59 responders (who all belonged to the terminal group) (23%) [Figure 2]. There were 12/59 failures and all these 12 patients belonged to the intermediate group (*n* = 26). Thus, nearly 50% of the patients in the intermediate group were failures, while there were no failures in the terminal group [Figure 2].

Adverse effects (calculated from all 354 treatment sessions) were limited to mild to moderate treatment pain in 100% of the treatment sites, short-term erythema in 90% (318), perfollicular edema in 80% (283), and blistering in 0.8% (3) of the patients. No incidence of hyper- or hypopigmentation was noted.

## DISCUSSION

Consistent with our study, many articles have suggested that multiple laser treatment sessions yield more effective results.[1–9] But only a few of them have commented upon multiple sessions (more than five) in darker skin types.[2,4,8,9] The findings reported in this study demonstrate that the long-pulsed Nd: YAG laser can be used safely and effectively for up to six multiple laser consecutive sessions in darker skin types [Figure 5A and 5B].

**Figure 5A:**
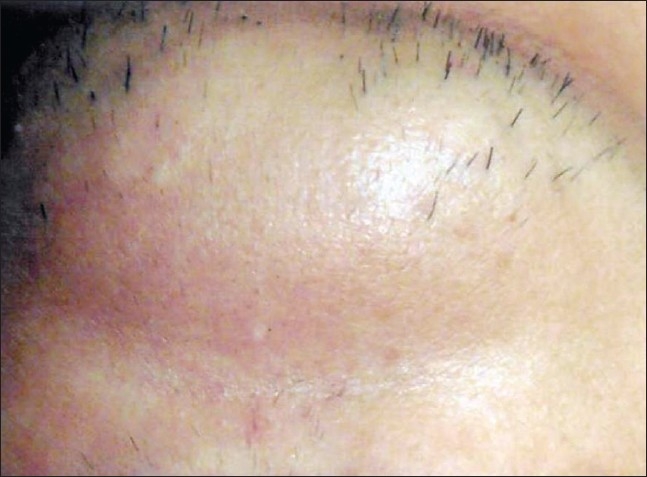
Grade 4 hair on chin in skin phototype IV before treatment

**Figure 5B:**
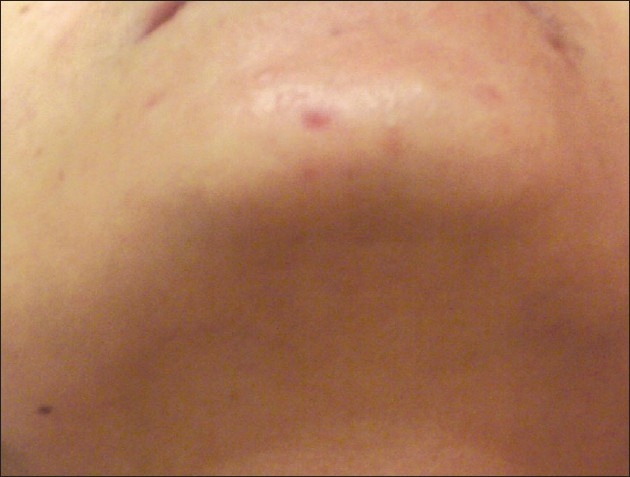
Grade 1 hair 6 weeks after 6 laser treatment sessions

Multiple sessions undoubtedly yield more effective results because lasers can only target the anagen or active phase of the hair growth cycle. At any given time, only 50–65% of facial hair is in the anagen phase for a duration of 3–4 weeks. Therefore, even if 100% of all anagen hairs are destroyed after each treatment, only a percentage of the total hair would be eliminated.[12] The same holds true for each successive treatment and hence, multiple treatments are required to achieve the best case scenario. An overall 56% hair reduction was reported in our study after a series of six treatment sessions with follow-up at six weeks. Previous studies with the Nd: YAG laser in skin phototypes IV–VI have shown a hair reduction varying from 23 to 33% after a single session,[1,2,3,14] 58 to 62% after three sessions[13] and 50 to 100% after 5–7 sessions[4,8,9] respectively at 4–12 weeks follow-up.

Studies with longer follow-up periods have shown a reduction varying from 41–46% after three sessions at six months,[15] 50–55% after three sessions,[1] and 35% after six sessions at 12 months’ follow-up.[2] The corresponding figures reported in our study seem to be lower than some of the aforementioned data. This is not unexpected as these have been conducted by different investigators using different grading and counting techniques making the comparison difficult. To date, hair counting is the most commonly used technique for assessing hair reduction, which unfortunately could not be performed in all cases in our study due to poor quality photographs. This is a limitation in our study. However, the our grading system had the advantage of providing the same yardstick of measurement (grade 1 hair) as a criterion for successful hair reduction to all the patients, irrespective of their baseline hair.

Melanin content and pulse duration are two important criteria for achieving effective hair reduction with lasers. Effective hair reduction is possible if the melanin within the hair shaft, hair follicle epithelium, and the heavily pigmented matrix, and also the stem cells in the area of the bulge, are targeted.[12,15] Only lasers with wavelengths ranging from 630 to 1100 nm can irradiate the entire length of anagen hair extenging 2–5 mm into the dermis.[1] In addition, pulse duration serves as an important parameter for effective photoepilation which should be shorter than or equal to the TRT of the hair shaft.[16] Both melanin content and TRT increase as the diameter of the hair shaft increases. A terminal hair has an average diameter of 60–80 µm. Vellus hairs are < 40 µm in diameter and contain little melanin, while the intermediate hair would fall somewhere in between.[10,12]

Therefore, for intermediate or a finer hair, a shorter TRT would be required which is not fully safe in darker skin, as it might be insufficient for an optimum cooling of the epidermis. In such a scenario, it is quite understandable that it is far easier and safer to remove a terminal hair than an intermediate hair, and even less so, a vellus hair. This is one of the reasons that most laser hair reduction studies have been performed on dark terminal hair only.

The laser sessions were generally well tolerated with minor side effects consistent with previous studies on the Nd: YAG laser.[1,2,13,15] No incidence of dyspigmentation or cutaneous textural changes were noted. Blistering was encountered in only three cases of intermediate hair type out of a total of 26 where high fluence and shorter pulse width had to be used to target the finer hair having shorter TRT as mentioned above. Most of the studies report blistering as an infrequent side effect,[4,15] while Bouzari *et al*.[8] reported it to be 18% and the second most common side effect after pain.

Based on the results of our study, it is possible to speculate that if more than six sessions had been given, many of the responders in our study might have been converted to achievers. However, inherent resistance to laser therapy cannot be ruled out in the case of failures. In such cases, it would be prudent to stop further treatment if no change is visible after six consecutive laser sessions. An important conclusion from our study is that terminal hair responds best whereas intermediate hairs are less likely to respond – a fact of which the patient should be informed during counseling. The chances of failure in thick hair are negligible whereas it is almost 50% in an intermediate hair. Likewise, there is also high probability of a thick hair to be a responder and a thin hair to be a failure.

## CONCLUSION

The results of this study support safe and effective use of six multiple consecutive laser sessions with long-pulsed, 1064 nm Nd: YAG laser for hair removal in Indian patients with darker skin photo types IV and V. The effectiveness of laser sessions was directly dependent upon the number of sessions.

The response to laser hair reduction varies depending on the type of hair. Dark, coarse terminal hair responds better than fine, low-density intermediate hair. However, larger studies are needed to further define the optimal number of treatment sessions and existence of a resistance pattern, if any, for each hair type as per our speculation.
